# Sex Specific Expression of Interleukin 7, 8 and 15 in Placentas of Women with Gestational Diabetes

**DOI:** 10.3390/ijms21218026

**Published:** 2020-10-28

**Authors:** Simon Keckstein, Sophia Pritz, Niklas Amann, Sarah Meister, Susanne Beyer, Magdalena Jegen, Christina Kuhn, Stefan Hutter, Julia Knabl, Sven Mahner, Thomas Kolben, Udo Jeschke, Theresa M. Kolben

**Affiliations:** 1 Department of Obstetrics and Gynecology, University Hospital, LMU Munich, Marchioninistr. 15, 81377 Munich, Germany; S.Pritz@gmx.de (S.P.); N.Amann@med.uni-muenchen.de (N.A.); Sarah.Meister@med.uni-muenchen.de (S.M.); Susanne.Beyer@med.uni-muenchen.de (S.B.); Magdalena.Jegen@med.uni-muenchen.de (M.J.); Hutter.Stefan@googlemail.com (S.H.); Julia.Knabl@gmx.de (J.K.); Sven.Mahner@med.uni-muenchen.de (S.M.); Thomas.Kolben@med.uni-muenchen.de (T.K.); Udo.Jeschke@med.uni-muenchen.de (U.J.); Theresa.Kolben@med.uni-muenchen.de (T.M.K.); 2 Department of Obstetrics and Gynecology, University Hospital Augsburg, Stenglinstr. 2, 86156 Augsburg, Germany; Christina.Kuhn@uk-augsburg.de; 3 Praxis PD Dr. Med. Stefan Hutter, Pferdemarkt 7, 94469 Deggendorf, Germany; 4 Department of Obstetrics and Gynecology, MVZ Hallerwiese, Johannisstraße 17, 90419 Nuremberg, Germany

**Keywords:** cytokines, gestational diabetes, placenta, trophoblast, decidua

## Abstract

Gestational diabetes mellitus (GDM) is known to increase the risk for feto-maternal complications during pregnancy. A state of low-grade inflammation, with elevated levels of proinflammatory molecules, similar to patients with obesity or diabetes mellitus type 2 has also been partly described in GDM. The placenta, as unique interface between mother and fetus, is not only passively affected by changes in one of these organisms, but also acts as a modulator by expressing hormones and cytokines. This study aimed to investigate the expression of the proinflammatory cytokines Interleukin (IL) 7, 8 and 15 in GDM in placental tissue. A total number of 80 placentas were included (40 GDM/40 control group). The expression of IL-7, 8 and 15 was investigated in extravillous trophoblast (EVT) and syncytiotrophoblast (SCT) by immunohistochemistry and immunofluorescence double staining. The immunohistochemical staining was evaluated with the semiquanitfied immunoreactive score (IRS). While the expression IL-15 was significantly upregulated in EVTs of women with GDM. The expression of IL-8 was significantly decreased in EVT of the GDM group. Furthermore, significant fetal sex specific differences were detectable in all three cytokines. Our findings suggest an involvement of the investigated cytokines in the maintenance of a state of chronic low-grade inflammation on placental level in patients suffering from GDM.

## 1. Introduction

Recent studies have shown that the prevalence of gestational diabetes (GDM) has increased over the past decades. This condition is increasing parallell to the rising prevalence of obesity and type 2 diabetes [1,2,3]. Women with gestational diabetes have a higher risk for complications during pregnancy such as the development of hypertension, preeclampsia and eclampsia, a higher incidence of cesarean sections and shoulder dystocia during delivery [4,5]. At the same time the fetus is at higher risk of suffering from diabetic fetopathy, placental malfunction and from postnatal hypoglycemia and acute respiratory distress syndrome [6,7]. Furthermore, elevated intrauterine glucose levels, due to diet-treated gestational diabetes or type 1 diabetes, increase the probability of developing type 2 diabetes in adult life [8].

Gestational diabetes is defined as impaired glucose intolerance primarily diagnosed in pregnancy or with onset in pregnancy [9,10]. GDM is based on the same risk factors as diabetes mellitus type 2, i.e., obesity, lack of exercise and genetic disposition. Additionally, the pathophysiology is suspected to be similar [11,12]. A state of low-grade inflammation, with elevated levels of proinflammatory molecules, could be detected in patients with obesity and is known to promote insulin resistance in diabetes mellitus type 2 [13,14]. Interestingly, the state of chronic inflammation in gestational diabetes is controversially discussed. While some studies report increased levels of proinflammatory molecules, others could not affirm these findings [15,16].

Due to its unique role as only interface between mother and fetus, the placenta is exposed to substrates, hormones and cytokines of both circulations. Furthermore, the placenta is not only passively affected by changes in one of these organisms, but also acts as a modulator by expressing hormones and cytokines [17,18]. Especially the last mentioned are pivotal for the placental function throughout pregnancy and play an important role in processes like villous formation and further differentiation [19]. Recent studies reported an overexpression of proinflammatory molecules in placentas of women with GDM [20]. This circumstance may promote the maintenance of low-grade inflammation and alter the differentiation process of the placenta throughout the pregnancy.

In our study the placental expression of the three cytokines IL-7, IL-8 and IL-15 was investigated. These cytokines are either known for their proinflammatory effect in association with obesity and gestational diabetes or have been associated with the implantation process of the placenta. For instance, IL-7 has been reportedly upregulated in adipose tissue of obese mice [21]. Apart from being secreted by lymphopoeitic tissue, IL-7 is also produced by a variety of cells including endothelial cells and expression can be stimulated through TNF-α [22,23]. Through high levels of glucose an upregulation of the secretion of IL-8 by endothelial cells has been observed and increased levels of IL-8 were detectable in visceral adipose tissue of women with GDM [24,25,26]. On the other hand, on the placental level, study results regarding IL-8 expression in GDM diverge and a positive correlation is not clear [14,25,27]. IL-15 plays an important role in the stimulation of uterine natural killer cells (NK). In a previous report our group could detect elevated levels of IL-15 in placentas of women with recurrent miscarriages [28]. In order to elucidate the rather unsatisfying knowledge regarding their role in GDM at the feto-maternal interface of the placenta, this study aimed to analyze the cytokine expression in placentas of diabetic mothers who delivered on term. A total of 80 Placentas were included (40 placentas of women with GDM/40 healthy women as a control group). To evaluate the respective interleukin expression in immunohistochemical staining, the semiquanitfied immunoreactive score (IRS) was used.

## 2. Results

### 2.1. Interleukin 7

#### 2.1.1. IL-7 Expression in Extravillous Trophoblasts (EVT)

**Figure 1:**
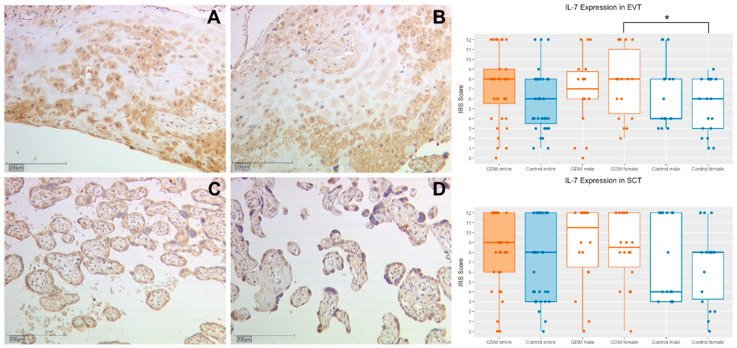
Representative immunohistochemical staining for IL-7 in extravillous trophoblast (EVT) in placentas of women with gestational diabetes mellitus (GDM) and the control group at a magnification of 10×. Staining of the EVT in a placenta affected by GDM (**A**) and of the control group (**B**). IL-7 expression of the syncytiotrophoblast (SCT) in placentas with GDM is demonstrated in image (**C**) and of the control group in image (**D**). The quantification of the IL-7 expression in placentas (with use of the IRS) of the study and control group and also their subgroups is shown in the box plots. Orange boxes represent the GDM group and blue boxes the control group. The range within the boxes represents the values between the 25th and 75th percentile with a horizontal line at the median. The T-bars extend 1.5 times of the interquartile range, or if no value is in that range, to the minimum or maximum value. The dots are marking the value of each data point. Significant results are linked with lines and marked with an asterisk (*p* < 0.05: *).

IL-7 expression could be identified in the EVT of placentas with gestational diabetes, as well as in those of the control group. The IL-7 expression was stronger in placentas with GDM in comparison to normal placentas even though without reaching statistical significancy (median IRS 8.0 vs. 6.0 *p* = 0.051, Figure 1). For further sex-specific analysis, placental tissue from female and male fetuses were compared to each other. No statistically relevant difference was found in the comparison of GDM placentas with normal placentas of male fetuses (median IRS 7.0 vs. 4.0; *p* = 0.461, Figure 1), the comparison of placentas from female fetuses showed a significant higher expression of IL-7 in the GDM group (median IRS 8.0 vs. 6.0; *p* = 0.028, Figure 1). The study groups internal, gender specific comparison of the IL-7 expression in the EVT did not reveal any significant difference (GDM male vs. female: median IRS 7.0 vs. 8.0, *p* = 0.704; control group male vs. female: median IRS 4.0 vs. 6.0; *p* = 0.206, Figure 1). A total of 78 placenta samples were investigated.

#### 2.1.2. IL-7 Expression in Syncytiotrophoblasts (SCT)

In syncytiotrophoblasts the staining of IL-7 was stronger in GDM placentas, but not statistically significant (IRS 9.0 vs. 8.0; *p* = 0.063, Figure 1 and Table 1). The gender-separated evaluation did not reveal any significant results (female GDM versus control: median IRS 8.5 vs. 8.0; *p* = 0.092; male GDM versus control: median IRS 10.5 vs. 4.0; *p* = 0.398, Figure 1 and Table 1). There were no group internal, gender specific differences (GDM male vs. female: median IRS 10.5 vs. 8.0; *p* = 0.341; control group male vs. female: 6.0 vs. 8.0; *p* = 0.263, Figure 1 and Table 1). A total of 80 placenta samples were investigated.

### 2.2. Interleukin 8

#### 2.2.1. IL-8 Expression in Extravillous Trophoblasts (EVT)

**Figure 2:**
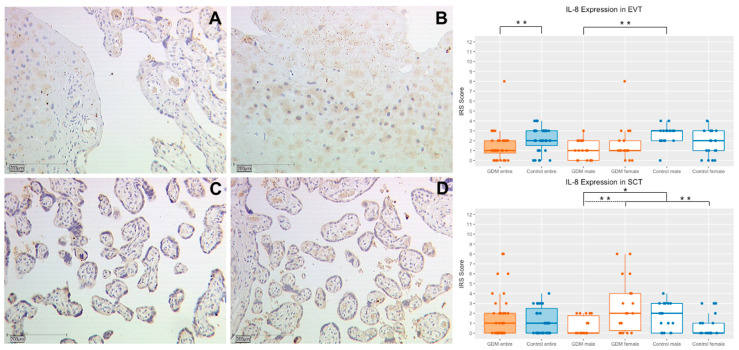
Representative immunohistochemical staining for IL-8 in EVT in placentas of women with GDM and the control group at a magnification of 10×. Staining of the EVT in a placenta affected by GDM (**A**) and of the control group (**B**). IL-8 expression of the SCT in placentas with GDM is demonstrated in image (**C**) and of the control group in image (**D**). The quantification of the IL-8 expression in placentas (with use of the IRS) of the study and control group and also their subgroups is shown in the box plots. Orange boxes represent the GDM group and blue boxes the control group. The range within the boxes represents the values between the 25th and 75th percentile with a horizontal line at the median. The T-bars extend 1.5 times of the interquartile range, or if no value is in that range, to the minimum or maximum value. The dots are marking the value of each data point. Significant results are linked with continuous or dashed lines and marked with an asterisk (*p* < 0.05: *; *p* < 0.01: **).

In extravillous trophoblasts of the GDM group, a significantly weaker expression of IL-8 in comparison to the control group could be detected (median IRS 1.0 vs. 2.0, *p* = 0.005, Figure 2 and Table 1). This result can also be transferred to the gender specific subgroups, even though the difference is only significant in the male subgroup (male GDM vs. control: median IRS 1.0 vs. 3.0, *p* = 0.001 female median IRS 1.0 vs. 2.0; *p* = 0.53, Figure 2 and Table 1). No significant difference in the expression of IL-8 in the EVT could be found in the group internal, gender specific analysis (GDM male vs. female: median IRS 1.0 vs. 1.0; *p* = 0.379; control group male vs. female: median IRS 3.0 vs. 2.0; *p* = 0.085, Figure 2 and Table 1). A total of 76 placenta samples were investigated.

#### 2.2.2. IL-8 Expression in Syncytiotrophoblasts (SCT)

While the general analysis showed no significant difference in the expression of IL-8 in the syncytiotrophoblast between the two study groups (median IRS 1.0 vs. 1.0; *p* = 0.516, Figure 2 and Table 1), the comparison of the gender specific subgroups revealed differences. GDM positive placentas from female fetuses showed a significantly higher expression of IL-8 (median IRS 2.0 vs. 0; *p* = 0.008, Figure 2 and Table 1). However, in placentas of male fetuses the IL-8 expression was higher in the control group (median 0 vs. 1.5; *p* = 0.042, Figure 2 and Table 1). The gender-specific group internal analysis revealed a significant higher IL-8 expression in the SCT of GDM placentas from female fetuses (median IRS 0.0 vs. 2.0; *p* = 0.004, Figure 2 and Table 1). No gender specific difference in the SCT was detected in the control group (median IRS male vs. female: 1.5 vs. 0 *p* = 0.074, Figure 2 and Table 1). A total of 80 placenta samples were investigated.

### 2.3. Interleukin 15

#### 2.3.1. IL-15 Expression in Extravillous Trophoblasts (EVT)

**Figure 3:**
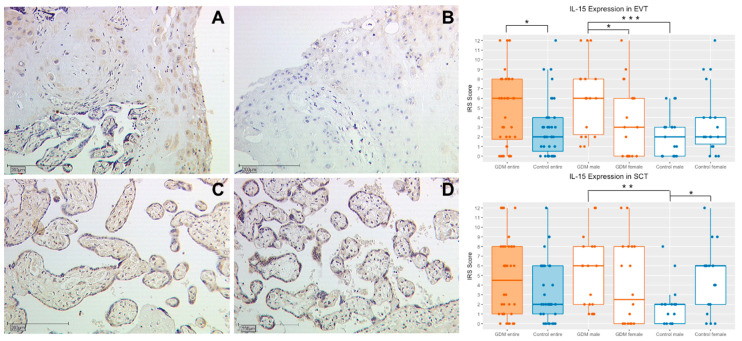
Representative immunohistochemical staining for IL-15 in EVT in placentas of women with GDM and the control group at a magnification of 10×. Staining of the EVT in a placenta with GDM is shown in image (**A**) and of the control group in image (**B**). IL-15 expression of the SCT in placentas with GDM is demonstrated in image (**C**) and of the control group in image **D**. The quantification of the IL-15 expression in placentas (with use of the IRS) of the study and control group and also their subgroups is shown in the box plots. Orange boxes represent the GDM group and blue boxes the control group. The range within the boxes represents the values between the 25th and 75th percentile with a horizontal line at the median. The T-bars extend 1.5 times of the interquartile range, or if no value is in that range, to the minimum or maximum value. The dots are marking the value of each data point. Significant results are linked with lines and marked with an asterisk (*p* < 0.05: *; *p* < 0.01: **; *p* < 0.001: ***).

Significant higher values of IL-15 could be measured in the EVT of GDM placentas in comparison to the control group (median IRS 6.0 vs. 2.0; *p* = 0.014, Figure 3 and Table 1). The result is also reproducible in the gender specific analysis, where IL-15 expression was significantly higher in the EVT of GDM placentas from male fetuses compared to the control group (median IRS 6 vs. 1; *p* < 0.001, Figure 3 and Table 1). The analysis of female placentas on the other hand could not reveal a difference (median IRS 3 vs. 2; *p* = 0.853, Figure 3 and Table 1). The group internal, gender specific analysis of the GDM group revealed a significant higher IL-15 expression in EVTs of male placentas compared to their female counterparts (median IRS 6 vs. 3; *p* = 0.026, Figure 3 and Table 1). There was no gender-specific difference of IL-15 expression in EVTs of the control group (median IRS 1 vs. 2, *p* = 0.125, Figure 3 and Table 1). A total of 78 placenta samples were investigated.

#### 2.3.2. IL-15 Expression in Syncytiotrophoblasts (SCT)

A not significant, nonetheless higher expression of IL-15 was detectable in the SCT of GDM placentas (median IRS 6.0 vs. 2.0; *p* = 0.096, Figure 3 and Table 1). The gender-separated analysis for female placentas could not reveal any difference in the expression of IL-15 in both study groups (median IRS 3.5 vs. 6.0, *p* = 0.649, Figure 3 and Table 1). For male placentas a significant higher expression of IL-15 was measurable in the GDM group (median IRS 6.0 vs. 2.0; *p* = 0.002, Figure 3 and Table 1). The direct comparison regarding the IL-15 expression in the SCT of male and female placentas with GDM did not show a significant difference (median IRS 6 vs. 3.5; *p* = 0.186, Figure 3 and Table 1). In the control group, significantly higher levels of IL-15 in the SCT could be detected in female placentas compared to male placentas (median IRS 2 vs. 6, *p* = 0.011, Figure 3 and Table 1). A total of 80 placenta samples were investigated.

### 2.4. Linear Median Regression Models for Interleukin Expression Including BMI and GDM

**Figure 4:**
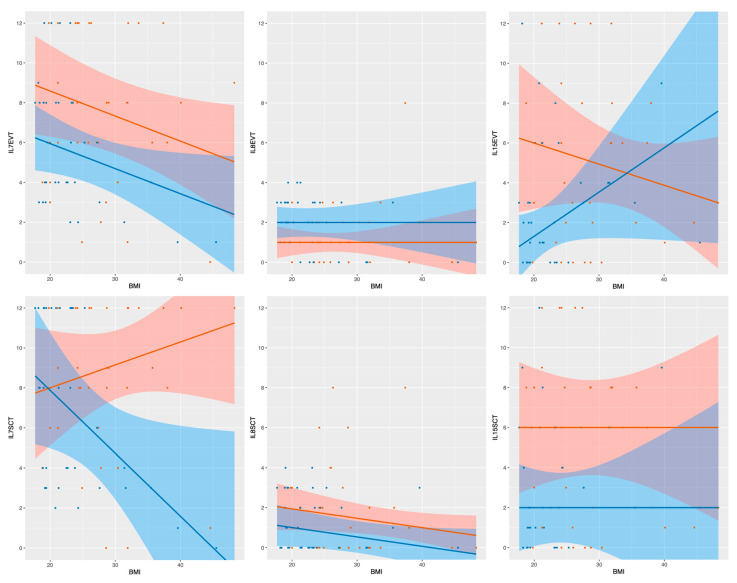
This figure shows the respective interleukin expression depending on being GDM positive or negative and the maternal BMI before pregnancy. The y axis shows the respective interleukin expression using the IRS. The x axis shows the BMI. The regression lines of each model of median regression are orange for the GDM positive group and blue for the GDM negative control group. The 95% confidence interval of each group is in the same colors. Every dot describes one case. In the plots of IL7 SCT and IL15 EVT the statistically significant interaction of both variables is shown. The remaining four figures show the interleukin expression depending on the BMI without interaction of those two variables.

Due to the significant higher BMI prior to pregnancy in the GDM group we investigated the influence of BMI regarding the interleukin expression in three models of median regression. The first model investigated the sole effect of GDM on the interleukin expression. The second model investigated the effect of GDM and BMI without interaction of those two variables, and the third model included the interaction of BMI and GDM. The model finally adopted is characterized by the property that addition of another variable or of an interaction term does not improve the model significantly. In the presence of main effects of both variables without interaction, the second model was chosen, and in the case of no significant effect of the BMI, the first model was adopted. In the following, not only the *p*-values are given, but also the estimated effect of the respective variable or of their interaction with each other (in regard to the IRS). For the variable GDM, the estimated effect describes the increase or decrease of the median interleukin expression in case of being GDM positive. Concerning BMI, the estimated effect describes the change in interleukin expression with the increase per BMI point. For a better illustration, the results are described in Table 2 and Figure 4.

### 2.5. Linear Median Regression Models for Interleukin Expression Including Birth Weight and GDM

Due to the significant higher birth weight in the GDM group, we also investigated the influence of birth weight regarding the interleukin expression in models of median regression. No significant interaction was detectable at all.

## 3. Discussion

The aim of this study was to investigate the placental cytokine expression in women with GDM in comparison to a control group. Additionally, potential sex-specific alterations and interactions with the BMI should be analyzed.

A tendency towards statistical significance with higher expression of IL-7 in GDM placentas in comparison to the control group could be found in the EVT (*p* = 0.051) and SCT (*p* = 0.063). The affiliation of the interleukin expression to either the EVT or the SCT was confirmed by double immunofluorescence staining.

As mentioned above, Interleukin 7 is known to be a proinflammatory cytokine and is mainly produced by stromal and vascular endothelial cells. IL-7 plays a role in the survival and proliferation of naive and memory B and T cells, mature T cells, and NK cells. The IL-7 receptor consists of the IL-7 specific IL7Rα (CD127) and the common gamma chain (χ_c_; Cd132) and is usually expressed by the aforementioned cells as well as dendritic cells and lymphoid tissue inducer cells. The expression of IL-7R is dynamically regulated by cytokines and by the general differentiation and metabolic state of the cells [29].

It is known that IL-7 is overexpressed in inflamed tissue, as well as in adipose tissue of obese women [22,30]. Furthermore, higher oxidative stress biomarkers in overweight women could be linked to an increased concentration of IL-7 in fetal blood after the delivery [31]. Interestingly, the analysis of a possible interaction of the maternal BMI with the IL-7 expression showed that a higher BMI does not lead to higher concentrations of IL-7. In the EVT the IL-7 expression declined with increasing BMI in both groups quite similar. In the SCT a significant interaction between BMI and both main groups were found. The IL-7 expression of the GDM group correlated positively the BMI, while in the control group the interleukin expression declined with an increasing BMI. These findings suggest a rather complex coherence between a state of chronic low-grade inflammation on a placental level and GDM, than just being a consequence of maternal obesity. Nevertheless, the higher expression of IL-7 in placentas of women in the GDM group supports the thesis of a proinflammatory environment in gestational diabetes.

TNF-α, as another proinflammatory cytokine, has been generally identified as an important factor for the maintenance of insulin resistance, and recent studies also detect higher TNF-α concentrations in placentas and amniotic fluid of women with GDM [17,32,33]. These findings may also be supported by the fact that TNF-α levels are declining after the delivery of the placenta, which is also accompanied by a reversal of the insulin resistance [19].

This aspect is of interest because the expression pattern of IL-7R has been reportedly upregulated by TNF-α and would support the thesis of the maintenance of an inflammatory environment through positive feedback.

Gender specific differences in the expression of immune mediators in amniotic fluid and placenta have been reported in the past, and Barke et al. could recently find a gender specific upregulation of proinflammatory genes in placentas of mice with GDM [34,35,36]. While some studies found that male placentas are more susceptible to changes in the maternal environment, other studies reported the opposite [37,38,39]. These findings suggest partly different coping strategies depending on fetal sex in environmental changes. Even though we could report a significant higher expression of IL-7 in EVT in female placentas compared to the female control group, the study groups internal, gender-specific comparison showed no significant difference.

IL-8 has been identified as a proinflammatory cytokine which is produced by a variety of cells including neutrophils, macrophages, monocytes, epithelial, and endothelial cells. The cytokine is associated with the activation and recruitment of neutrophils in infected or injured tissue, as well as in adipose tissue [29,40]. Furthermore, high levels of glucose are able to enhance the release of IL-8 from endothelial cells [24]. IL-8 has also been the subject of investigation regarding its role in gestational diabetes and obesity in pregnancy with varying results. For instance, studies could detect higher levels of IL-8 in visceral adipose tissue of women with GDM [25,26]. While Zhang et al. recently could demonstrate that serum levels of IL-8 significantly correlate with GDM, Kuzmicki was not able to support this thesis [41]. Regarding the expression of IL-8 on placental level, some studies, did not find any significant difference in comparison to control individuals [14,25] and other study results were rather inconclusive [27].

In our study IL-8 was detectable in both study groups, but in general with a rather weak expression. In extravillous trophoblasts of GDM placentas a significant weaker expression of IL-8 in comparison to the control group could be detected, which was mainly found in the group of male fetuses. While the general analysis of the SCT showed no significant difference in the expression of IL-8 between the two study groups, the gender specific analysis interestingly showed a diametrical result: in placentas from female fetuses the GDM group was associated with a significantly higher expression of IL-8. In male gender placentas however, the IL-8 expression was higher in the control group (although without significance). In multiple models of median regression, no influence of the maternal BMI on the interleukin expression was detectable.

The phenomenon of higher levels of IL-8 in female over male placentas due to other causes of chronic low-grade inflammation, such as mild asthma has been published in the past [42]. On the other hand, Muralimanoharan et al. found higher levels of IL-8 in male placentas in preeclamptic women in comparison to the female group [43]. Nonetheless, current data and our results suggest the presence of sexual dimorphism regarding the expression of IL-8 on placental level.

Due to the partly contrary results, a rather complex role of IL-8 in GDM must be assumed. In context with the relatively low expression rates, one could also suggest a rather subordinate role of IL-8 in the interplay of interleukins at the feto-maternal interface in GDM. However, changes of expression could be observed, so further studies of its role in GDM are definitely warranted.

Like IL-7 and IL-8, IL-15 has proven to be a proinflammatory cytokine, which can be produced by immune cells like monocytes or T cells, as well as nonimmune cells like skeletal muscle cells, keratinocytes or mucosal stromal cells [40,44]. IL-15 is known for its activation of T cells and plays a pivotal role in the stimulation and differentiation of uterine NK cells [40,45]. At the beginning of the pregnancy, those cells have been described as an important factor in the differentiating process of stromal cells into decidual cells and modifying spiral arteries [46]. The importance of IL-15 regarding the stimulation of NK cells was underlined by Barber, who demonstrated that deficiency of this specific cytokine results in an absence of NK cells [47]. Physiologically, NK cells accumulate during early pregnancy around trophoblast cells before decreasing in concentration during the following course of the pregnancy [44]. In GDM, where a hyperglycemic state generates a proinflammatory environment, Chiba et al. showed that the percentages of NK cells producing IFN-χ and TNF-α were significantly higher in women with GDM in comparison to normoglycemic individuals. Furthermore, Hara et al. could detect an increase of NK cells in term placentas in women with GDM compared to normoglycemic women [27]. Despite these results and the fact that IL-15 plays an important role in the differentiation of NK cells, the expression of IL-15 on the placental level has not been investigated before.

A significantly higher expression of IL-15 could be detected in the EVT of GDM placentas in comparison to the control group. IL-15 was also trending to a higher expression in the SCT of the GDM group, even without reaching significance. Furthermore, the analysis of multiple models of median regression showed a significant interaction of maternal BMI and the interleukin expression in the EVT, leading to a decrease in the staining intensity with increasing BMI in the GDM group and an opposite effect in the control group. Supporting the results of Hara et al., the elevated IL-15 expression might be responsible for an increased differentiation and stimulation of NK cells [27]. Moreover, elevated levels of TNF-α production in GDM placentas have been reported [32] and additionally an increase of TNF-α production of NK cells after stimulation with IL-15 was also described [48,49]. Supporting a possible concept of fetal sexual variation in placental cytokine expression, the group internal, gender-specific analysis in women with GDM revealed a significantly higher expression in the EVT of male placentas in comparison to the female ones. Furthermore, the gender specific analysis showed only significant higher expression of IL-15 in male placentas in EVT and SCT of women with GDM in comparison to the control group, while no levels of significance were detectable in female placentas.

In conclusion, our results provide new evidence of an involvement of the investigated interleukins in the process of gestational diabetes with sex-specific alterations. They seem to create and support a proinflammatory environment on a placental level in GDM.

Yet, the complex interplay of these cytokines at the feto-maternal interface, as well as gender-related variations, cannot be considered understood.

## 4. Materials and Methods

### 4.1. Tissue Samples

In this study a total number of 80 placentas from the Department of Obstetrics and Gynecology Ludwig-Maximilians-University Munich were included. 40 placentas were obtained from women who were diagnosed with gestational diabetes. The other 40 placentas were obtained from women with uncomplicated pregnancies. All patients were informed about the study and gave their written consent. The study was approved by the ethical committee of the University of Munich (approved amendment for project 337-06, on the 26 January 2010).

The inclusion criteria for the GDM group was a diagnosed with gestational diabetes mellitus of the mother in concordance to the WHO definition with at least one pathologic oGTT parameter between the 24th and 28th week of gestation. Women of the control group had to have non-pathological oGTT parameters. Both groups were subdivided into two subgroups regarding the sex of the fetus. Each of the four groups contained a total of 20 placentas. Exclusion criteria were multiple birth, premature delivery, intrauterine growth restriction (IUGR), fetal malformation and infection. Demographic and clinical data of the study population are shown in Table 3.

Immediately after vaginal delivery or caesarean section, the tissue samples were taken from the central part of the placenta and fixed in 4% Formalin. After 24 h they were embedded in paraffin for the immunohistology. In the next step, the samples were cut with a sliding microtome to 2–3 µm slices.

### 4.2. Immunohistochemistry

For the next step, the paraffin sections had to be deparaffinized with xylol and afterwards bathed in 100% ethanol. To stop the endogenous peroxidase activity, the samples were incubated for 20 min in methanol/H_2_O_2_ and rehydrated in alcohol gradient to distilled water. Slices which were later incubated with antibodies against IL-7 and IL-8 were put in a high pressure cooker for 5 min using sodium citrate buffer (pH 6.0) for antigen retrieval. This step was not necessary for slices which were incubated with antibodies against IL-15. All antibodies which were used in this study are listed in Table 4.

In the next step, the slices were treated for 3 min with a power block for saturating electrostatic charges. Then tissue sections were incubated for at least 16 h at 4 °C with the respective primary antibody (against IL-7, 8 or 15). After washing the slides with phosphate-buffered saline (PBS), the Vectastain Elite ABC mouse-IgG-Kit (Vector Laboratories, Burlingame, USA) was used for the visualization. The slices were counterstained with Mayer’s acid hemalum for 2 min and stained blue for 5 min in water. In the following step the samples were dehydrated in an ascending series of alcohol, then treated with xylol and cover-slipped with Shandon Consul-Mount (Thermo Fisher Scientific, Waltham, USA).

For the evaluation of the intensity and distribution patterns of the antigen expression the semiquantitative immunoreactive score of Remmele (IRS) was used [50]. The IRS is calculated by the multiplication of the grade of optical staining intensity (0 = none, 1 = weak, 2 = moderate and 3 = strong staining) and the percentage of positive staining cells (also divided into 4 categories: 0 = no staining, 1 = 1–10% of the cells, 2 = 11–50% of the cells, 3 = 51–80% of the cells and 4 = more than 80% of the cells).

### 4.3. Double Immunofluorescence Staining

Double Immunofluorescence staining allows us to characterize specific antigens simultaneously. In this study we wanted to assign each of the investigated interleukins exemplary to the extravillous trophoblast (EVT) or syncytiotrophoblast (SCT). For the evaluation, slides from tissue blocks of both study groups were used. Due to the characteristic expression of human leukocyte antigen (HLA), and in cells of the EVT, HLAG-FITC antibodies were used.

In the first step, slides had to be deparaffinized with xylol and rehydrated in alcohol gradient to distilled water. To achieve an antigen retrieval slides had to be put in a high-pressure cooker for 5 min using sodium citrate buffer (pH 6,0). Subsequently, a 15-min incubation with Ultra Vision protein block was accomplished to prevent an unspecific staining.

**Figure 5:**
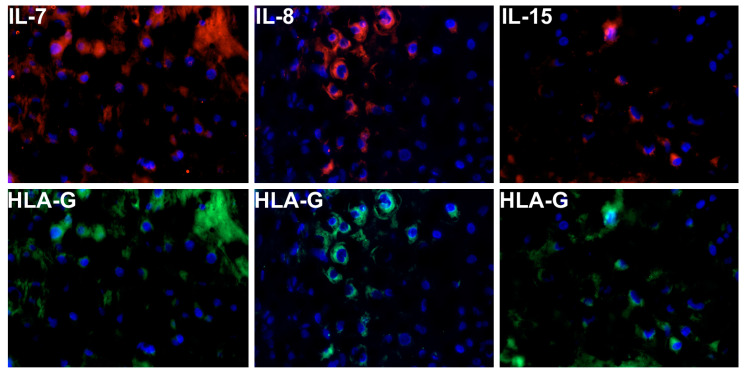
Double immunofluorescence staining was used for the simultaneous proof of the respective interleukin and HLA expression in cells of EVT. Cy3 marked antibodies (red staining) are showing the respective interleukin expression (first line of the figure). HLA-G antibodies (FITC marked) were used to picture the EVT in green (second line of the figure). Cell nuclei are stained with DAPI in blue.

Overnight, the slides were incubated with die respective primary antibodies against IL-7 (Dilution 1:100), IL-8 (Dilution 1:250) and IL-15 (Dilution 1:1000) at 4 °C. As secondary antibody, Cy3 marked goat-anti-mouse IgG (dilution: 1:500) was added and incubated for 30 min at room temperature. After washing the slides with PBS, the samples were incubated for one hour with the HLAG-FITC antibody to mark the EVT and afterwards again washed with PBS. Until slides were dried, they were stored in the dark and then embedded in mounting buffer, which contained 4′,6-diamino-2-phenylindole for blue staining of the nucleus. The slides were examined with a Zeiss Axiopphotomicroscope (Zeiss, Jena, Germany). Images were generated with a digital camera system Axiocam (Zeiss, Jena, Germany). Example images are shown in Figure 5.

### 4.4. Statistics

The statistical programming environment R, version 4.0.2, was used for processing and statistical analysis of the collected data [51]. To compare distributions of interleukin scores across groups we applied non-parametric *t*-tests, which do not assume normality or shift invariance of the considered distributions [52]. *p*-values were not adjusted in multiple comparisons. Similarly, since error terms in usual linear mean regression models turned out to be non-normally distributed, we chose linear median regression models, which themselves are more robust against outliers [53]. Furthermore, Welch’s *t*-test and chi-square test were used. Findings with *p*-values < 0.05 were considered significant.

## Figures and Tables

**Table 1:** This table shows the median IRS values for both main groups and each subgroup in EVT and SCT. Furthermore, the respective interquartile range is indicated in the brackets.

		GDM Entire	Control Entire	GDM Male	GDM Female	Control Male	Control Female
IL-7	EVT	8.0 (3.5)	6.0 (4.5)	7.0 (3.75)	8.0 (6.5)	4.0 (4.0)	6.0 (5.0)
	SCT	9.0 (6.0)	8.0 (9.0)	10.5 (5.5)	8.5 (5.5)	4.0 (9.0)	8.0 (4.75)
IL-8	EVT	1.0 (1.25)	2.0 (1.5)	1.0 (2.0)	1.0 (1.0)	3.0 (1.0)	2.0 (2.0)
	SCT	1.0 (2.0)	1.0 (2.5)	0.0 (1.75)	2.0 (3.75)	2.0 (3.0)	1.0 (1.0)
IL-15	EVT	6.0 (6.25)	2.0 (3.5)	6.0 (5.75)	3.0 (6.0)	2.0 (3.0)	2.0 (2.75)
	SCT	4.5 (7.0)	2.0 (5.0)	6.0 (6.0)	2.5 (8.0)	2.0 (2.0)	6.0 (4.0)

**Table 2:** Linear Median Regression Models for Interleukin Expression Including BMI and GDM.

Outcome Variable	Input Variable in Model of Median Regression	*p*-Values in F-Statistics When Comparing Model with Previous Model Using ANOVA	Coefficients	Estimate	Std. Error	*p*-Values
IL7 EVT	GDM	*p* < 0.001 ***	GDM pos.	1.00000	0.64017	0.122
	**GDM+BMI**	*p* < 0.001 ***	GDM pos.	1.32075	0.58290	0.026 *
			BMI	−0.12579	0.06127	0.043 *
	GDM + BMI + GDM:BMI	0.067	GDM pos.	−1.60090	1.93476	0.410
			BMI	−0.11211	0.06038	0.067
			GDM pos.:BMI	0.11211	0.06038	0.067
IL7 SCT	GDM	0.010 *	GDM pos.	0.50000	0.87660	0.570
	GDM+BMI	0.071.	GDM pos.	1.95906	0.90459	0.0336 *
			BMI	−0.29240	0.14451	0.046 *
	**GDM + BMI + GDM:BMI**	0.023 *	GDM pos.	−4.23119	2.69147	0.120
			BMI	−0.09976	0.09253	0.284
			GDM pos.:BMI	0.21470	0.09253	0.023 *
IL8 EVT	**GDM**	*p* < 0.001 ***	GDM pos.	−0.50000	0.20080	0.015 *
	GDM+BMI	0.594	GDM pos.	−0.50000	0.22848	0.032 *
			BMI	0.00000	0.03716	1.000
	GDM + BMI + GDM:BMI	0.330	GMD pos.	−1.59677	0.95722	0.099
			BMI	−0.04608	0.04581	0.318
			GDM pos.:BMI	0.04608	0.04581	0.318
IL8 SCT	**GDM**	0.027 *	GDM pos.	0.00000	0.32031	1.000
	GDM+BMI	0.054.	GDM pos.	0.46714	0.28318	0.103
			BMI	−0.04695	0.02689	0.085
	GDM + BMI + GDM:BMI	0.434	GDM pos.	0.91727	0.84417	0.280
			BMI	−0.05591	0.02278	0.016 *
			GDM pos.:BMI	−0.01789	0.02278	0.434
IL15 EVT	**GDM**	0.015 *	GDM pos.	2.00000	0.64008	0.002
	GDM+BMI	0.122	GDM pos.	2.00000	0.70699	0.006
			BMI	0.00000	0.08691	1.000
	**GDM + BMI + GDM:BMI**	0.043 *	GDM pos.	5.62674	2.24393	0.014 *
			BMI	0.05811	0.07962	0.468
			GDM pos.:BMI	−0.16411	0.07962	0.043 *
IL15 SCT	**GDM**	0.144	GDM pos.	2.00000	0.76082	0.010 *
	GDM+BMI	0.262	GDM pos.	2.00000	0.77757	0.012 *
			BMI	0.00000	0.10292	1.000
	GDM + BMI + GDM:BMI	0.107	GDM pos.	5.69877	2.88469	0.052
			BMI	−0.00362	0.09952	0.971
			GDM pos.:BMI	−0.16235	0.09952	0.107

This table shows the results of the three models of linear median regression investigating the influence of the BMI regarding the interleukin expression. The first model investigates the sole effect being GDM on the interleukin expression. The second model investigates the effect of BMI and GDM without interaction of those variables, and the third model includes the interaction of BMI and GDM. The third column shows the *p*-value of this particular model compared to the previous less complex model. The choice of the ultimately chosen model depends on this value. The first model (GDM only) is compared to the respective mean of the interleukin expression. The column “estimate” shows the effect on each variable in changing the related input variable(s) by one unit. The column “standard error” refers to the estimation error in the effect estimate in the previous column. The *p* value given in the seventh column is related to the null hypothesis of no effect of the respective variable. The gray-shaded rows with variables in bold show the models of median regression which describes the data best. Significant results are linked with lines and marked with an asterisk (*p* < 0.05: *; *p* < 0.001: ***).

**Table 3:** Demographic and clinical characteristics of the study population. The values are listed as mean ± SD. Significant *p*-values are written in bold.

	GDM	Control Group	*p*-Value
Maternal age (years)	32.83 ± 4.56	31.15 ± 6.10	0.165
Body mass index (BMI)	28.13 ± 6.96	23.35 ± 6.21	**0.002**
Gestational age (weeks)	39.85 ± 1.29	39.78 ± 1.35	0.800
Birth weight (g)	3611.38 ± 0.08	3316.88 ± 501.73	**0.013**
pH umbilical artery	7.30 ± 0.08	7.29 ± 0.09	0.587
Vaginal birth (%)	67.5	79.5	0.171
Contractions (%)	85	82.5	0.727

**Table 4:** This table shows the antibodies used for immunohistochemical characterization in this study.

Antibody	Isotype	Clone	Dilution	Source
Goat-Anti-Mouse Cy3	Goat IgG	Polyclonal	1:500	Dianova, Hamburg Germany
HLA-G (FITC marked)	Mouse IgG	Monoclonal	1:100	Bio-Rad Laboratories, Hercules, USA
IL-15	Mouse IgG	Monoclonal	1:1000	Abcam, Cambridge, UK
IL-8	Mouse IgG	Monoclonal	1:250	Abcam, Cambridge, UK
Il-7	Mouse IgG	Monoclonal	1:100	R&D Systems, Minneapolis, USA

## Abbreviations

GDM	Gestational diabetes mellitus
IL	Interleukin
EVT	Extravillous trophoblast
SCT	Syncytiotrophoblast
IRS	Immunoreactive score
NK	Natural killer cells
PBS	phosphate-buffered saline
